# Methylated DNA is over-represented in whole-genome bisulfite sequencing data

**DOI:** 10.3389/fgene.2014.00341

**Published:** 2014-10-21

**Authors:** Lexiang Ji, Takahiko Sasaki, Xiaoxiao Sun, Ping Ma, Zachary A. Lewis, Robert J. Schmitz

**Affiliations:** ^1^Department of Genetics, University of Georgia, Athens, GAUSA; ^2^Institute of Bioinformatics, University of Georgia, Athens, GAUSA; ^3^Department of Microbiology, University of Georgia, Athens, GAUSA; ^4^Department of Statistics, University of Georgia, Athens, GAUSA

**Keywords:** DNA methylation, whole genome bisulfite sequencing, Epigenomics, epigenetics, PCR bias

## Abstract

The development of whole-genome bisulfite sequencing (WGBS) has resulted in a number of exciting discoveries about the role of DNA methylation leading to a plethora of novel testable hypotheses. Methods for constructing sodium bisulfite-converted and amplified libraries have recently advanced to the point that the bottleneck for experiments that use WGBS has shifted to data analysis and interpretation. Here we present empirical evidence for an over-representation of reads from methylated DNA in WGBS. This enrichment for methylated DNA is exacerbated by higher cycles of PCR and is influenced by the type of uracil-insensitive DNA polymerase used for amplifying the sequencing library. Future efforts to computationally correct for this enrichment bias will be essential to increasing the accuracy of determining methylation levels for individual cytosines. It is especially critical for studies that seek to accurately quantify DNA methylation levels in populations that may segregate for allelic DNA methylation states.

## INTRODUCTION

The ability to produce genome-wide data sets has radically changed during the last decade. With the advent of high-throughput sequencing, billions of short read data can be generated within days. Currently, one of the primary uses of these short reads is to explore genetic variation within the human population with the goal of linking variants to diseases. Identifying these causal associations is the first major step to understanding how diseases arise, how they manifest and how they can be treated. Additionally, these short reads are used to study a wide range of model systems and an ever-growing list of non-model systems. Although much of the energy in the scientific community focuses on genetic variants there are a growing number of scientists that are fascinated by the epigenome. The NIH Roadmap Epigenomics Mapping Consortium defines the epigenome as a map of features distributed throughout and on top of the genome (Bernstein et al., 2010). These features can include RNAs such as mRNAs, long non-coding RNAs and small RNAs, DNA-protein interactions, chromatin accessibility, nucleosome positioning, covalent modifications to histone tails and DNA methylation, to name a few. Understandably, refinement of epigenomic techniques lagged behind development of genomic DNA library preparation due to their demand for use and due to the difficulty in execution. Part of the reason for this sudden increase and interest in epigenomics is due to the standardization of protocols and analysis pipelines used to produce and interpret data. No longer is data generation the rate-limiting step. Instead, the ability to accurately analyze and interpret these data sets has become the major bottleneck in the daily workflow.

One of the most exciting technical advances in field of epigenomics was the development of methods to analyze DNA methylation at single-base resolution (Cokus et al., 2008; Lister et al., 2008). Generally referred to as whole-genome bisulfite sequencing (WGBS), these methods combine sodium bisulfite conversion (Hayatsu et al., 1970; Shapiro et al., 1973, 1974; Hayatsu, 1976; Frommer et al., 1992; Clark et al., 1994, 2006), the gold standard for determining DNA methylation states at individual cytosines, with high-throughput DNA sequencing. Using this technique, unmethylated cytosines are converted into uracils and then into thymines after PCR. The observation of cytosines in sequencing reads typically indicates that the cytosine was methylated, as methylcytosines are protected from conversion by treatment with sodium bisulfite. These methods are capable of testing approximately 90% of all cytosines in genomes studied to date. They also generate quantitative measurements of DNA methylation at each cytosine due to the multiple independent reads that align to a particular sequence. These methods were originally tested on *Arabidopsis thaliana* genomic DNA (Cokus et al., 2008; Lister et al., 2008) as this species has a relatively small genome size (∼150 Mb) compared to human genomes (∼3 Gb per haploid genome), which made testing and troubleshooting cost efficient. Since the first single base resolution DNA methylomes for *A. thaliana* surfaced, larger DNA methylomes have been sequenced such as humans (Lister et al., 2009), mice (Stadler et al., 2011), corn (Gent et al., 2013), and soybean (Schmitz et al., 2013a), but in general it is cost prohibitive to sequence large numbers of individual samples using this technique. As a result, reduced representation bisulfite sequencing (RRBS) was developed in which ∼1% of the genome is studied making it possible to investigate large numbers of individuals at the cost of surveying the entire genome (Meissner et al., 2005). Most recently, additional modifications to cytosines have been identified such as 5-hydroxymethylation, 5-formylcytosine, and 5-carboxylcytosine and methodologies have been developed to study the presence of some of these base modifications genome-wide (Booth et al., 2012, 2014; Yu et al., 2012).

The initial genome-wide maps of DNA methylation were invaluable for defining previously unexpected epigenomic signatures in a variety of systems, such as partially methylated domains and non-CG methylation in animals (Lister et al., 2009) and CHH islands in maize (Gent et al., 2013). Since these initial maps, WGBS has been used to study epigenome reprogramming of induced pluripotent stem cells (Lister et al., 2011), genomic imprinting in both plants (Gehring et al., 2009; Hsieh et al., 2009; Zemach et al., 2010; Calarco et al., 2012) and animals (Xie et al., 2012; Kobayashi et al., 2013; Shirane et al., 2013), germ line epigenome reprogramming in plants (Slotkin et al., 2009; Calarco et al., 2012; Ibarra et al., 2012; Creasey et al., 2014) and animals (Seisenberger et al., 2012; Jiang et al., 2013) and also to study the impact of genetic variation on DNA methylation (Schmitz et al., 2013a,b; Orozco et al., 2014). Clearly, mapping and analyses of DNA methylomes is a relatively young field with great potential for exciting discoveries in the next decade.

It is well established that there is a GC content bias in amplification of fragments of DNA used for high-throughput sequencing applications (Aird et al., 2011; Benjamini and Speed, 2012). As a result, methods were developed to avoid PCR amplification all together in the construction of DNA sequencing libraries (Kozarewa et al., 2009). However, this approach is not applicable to bisulfite-treated DNA as the uracils in the fragments would inhibit cluster formation on the instrument unless reagents could be customized to include a uracil-insensitive DNA polymerase. As a result, bisulfite-treated DNA is amplified for varying cycles of PCR to replace uracils in the DNA sequence and to amplify libraries as treatment with sodium bisulfite degrades DNA. The degradation of DNA by sodium bisulfite also creates limitations with regards to the types of samples that can be sequenced, as higher input amounts are often required for most protocols. Moreover, after bisulfite conversion, previously complementary strands are single stranded and will no longer anneal together. In fact, previous observations of bisulfite-conversion and PCR have uncovered strand biases that severely affects estimates of DNA methylation levels (Warnecke et al., 1997). Correction methods have been developed for targeted bisulfite PCR experiments, but these methods are not feasible for WGBS (Komori et al., 2011; Moskalev et al., 2011). Given that methylated DNA will retain higher GC content after bisulfite conversion and PCR compared to unmethylated DNA, over-representation of methylated DNA may occur in the construction of sequencing libraries. Although libraries can be constructed from lower input DNA samples such as those prepared from FFPE (formalin-fixed paraffin-embedded), sequence-capture or single cell approaches, there usually is a cost of increasing the total PCR cycles. This cost likely comes in the form of increasing any bias in read enrichment between methylated and unmethylated sequences at the same locus.

In this study, we explore the presence of an amplification bias created in fragments that retain higher GC content after sodium-bisulfite treatment with unmethylated sequences and with genomic DNA sequences. A strong positive correlation between DNA methylation levels and normalized read counts are observed in the WGBS data. Investigation of three different commercially available uracil-insensitive enzymes reveal differences in amplification bias of highly methylated and high GC content DNA sequences.

## MATERIALS AND METHODS

### PLANT AND FUNGAL MATERIAL

The *A. thaliana* Col-0 accession was used for all. Plants were grown in 16 h day lengths and tissue was harvested from above ground rosette leaves 3 weeks after planting. DNA was isolated using the Qiagen (Valencia, CA, cat-69106, USA) DNeasy Plant Kit according to the manufacturer’s protocol. All *Neurospora* experiments were performed with the wild-type “Oak Ridge” strain (FGSC# 2489) obtained from the Fungal Genetics Stock Center (McCluskey et al., 2010). ∼1 × 10^6^ conidia/ml were germinated in 50ml of Vogel’s Minimal Medium with 1.5% sucrose and grown for 5 h.

### METHYLC-SEQUENCING LIBRARY PREPARATION

MethylC-seq libraries were prepared according to the following protocol (Urich et al., 2014), with the some modifications. Three different enzyme mixtures were used to amplify bisulfite-converted adapter ligated DNA: Kapa HiFi Uracil+ (cat-KK2802, Kapa Biosystems), Pfu Turbo Cx Hotstart DNA polymerase (Agilent Technologies, Santa Clara, CA, cat-600410, USA), and EpiMark (New England Biolabs., Ipswich, MA, cat-M0490S, USA). PCR cycle number varied from 4, 8, or 15 cycles as described in the main text.

### PREPARATION AND SEQUENCING OF *Neurospora* DNA

Chromatin immunoprecipitation (ChIP) and DNA isolation were performed as described previously (Sasaki et al., 2014). For Illumina sequencing, libraries were prepared from 10 ng of ChIP input DNA using an Illumina TruSeq kit (Illumina cat-FC-121-2002). Libraries were prepared according to manufacturer instructions except for the following modifications. DNA adaptors were diluted 1:100 prior to ligation. PCR was performed for 4, 8, or 15 cycles, as indicated in the text. In some experiments, a PCR reaction mixture containing Kapa HiFi polymerase (Kapa Biosystems, cat-KK2502), 1X Kapa HiFi buffer, 200 nM dNTPs, 1X TruSeq primer cocktail, and 60 mM tetramethylammonium chloride (TMAC; Sigma-Aldrich, catalog # T3411) was used in place of the TruSeq PCR master mix [as described in (Oyola et al., 2012)]. Illumina sequencing was performed using an Illumina Next-Seq500 instrument at the University of Georgia genomics facility.

### METHYLC-SEQ DATA ANALYSIS

FASTQ files were trimmed for adapters, preprocessed to remove low quality reads and aligned to the TAIR10 reference genome as previously described in (Schmitz et al., 2013b). Inefficiencies in the sodium bisulfite conversion reaction are calculated by measuring the fraction of methylated basecalls detected in the chloroplast genome (which is unmethylated). This non-conversion rate is used as the null hypothesis for a binomial test to determine if a cytosine is methylated and the resulting *p*-values are corrected for multiple testing using Benjamini–Hochberg with an FDR cut off of 5%.

### CALCULATION OF METHYLATION LEVELS

Weighted methylation levels were performed as described in (Schultz et al., 2012).

### CALCULATION OF CORRELATION COEFFICIENTS

Pearson correlation was calculated. In **Figure 2B**, the outliers were identified using Bonferroni test (Cook and Weisberg, 1982) and then Pearson correlation was calculated after the outliers were removed.

### CALCULATION OF NORMALIZED READ COVERAGE

**FIGURE 1:**
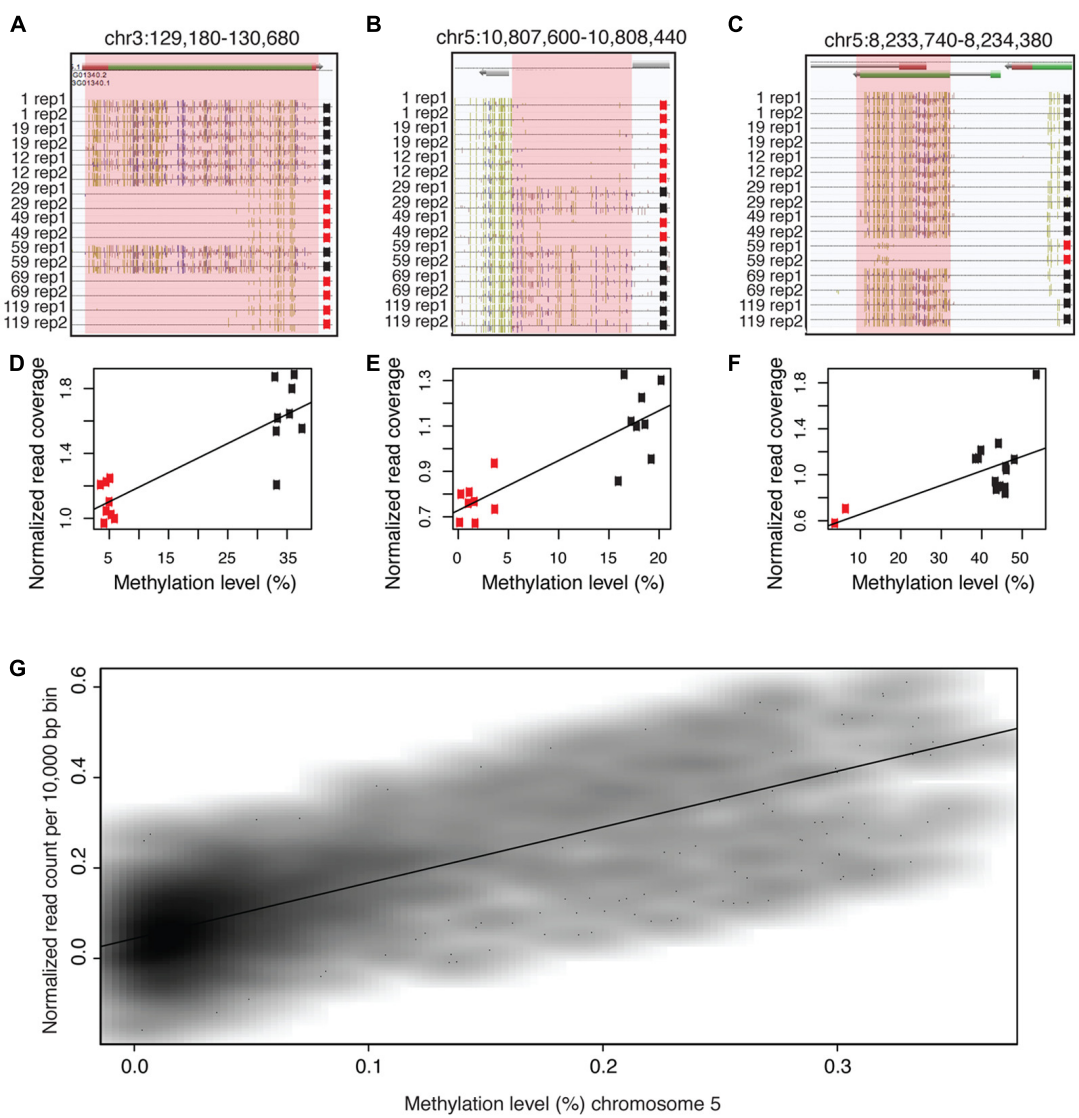
**Methylated DNA is enriched in **whole-genome bisulfite sequencing data (WGBS)**. (A–C)** Examples of differentially methylated regions (DMRs) that contain identical DNA sequences. **(D–F)** Highly methylated regions contain an abundance of bisulfite sequencing reads compared to lowly methylated sequences. **(G)** Highly methylated DNA is positively correlated with bisulfite sequencing read counts. The names of the samples in **(A–C)** indicate lineage numbers as previously reported Shaw et al. (2000). Pink shaded regions indicate DMRs in **(A–C)** and the location of data used to produce **(D–F)**. In **(A–C)**, gold lines = methylated CGs, blue lines = methylated CHGs (whereH = A, C, or T) and pink lines = methylated CHHs. In **(D–F)**, black dots = highly methylated alleles, red dots = lowly methylatedalleles. Data used to produce **Figure 1G** were obtained from line1 replicate 2 from (Schmitz et al., 2011).

**FIGURE 2:**
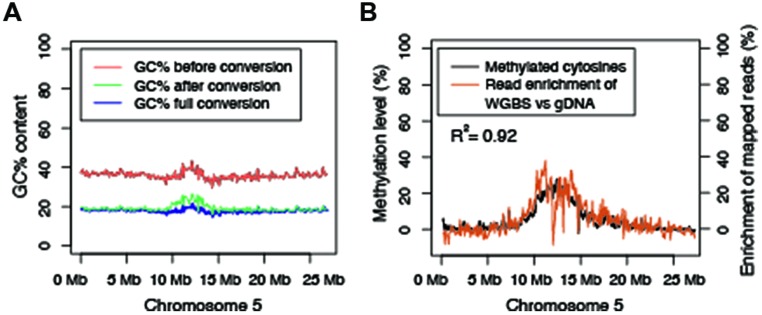
**Methylated DNA has a skewed GC content after sodium bisulfite conversion of DNA and this leads to an enrichment of methylated reads in bisulfite sequencing. (A)** GC content beforebisulfite conversion, after bisulfite conversion, computed full conversion and the absolute difference in GC content between unconverted and bisulfite-treated DNA. **(B)** WGBS reads are positively correlated and enriched with highly methylated regions of the chromosome compared to alignment of sequencing reads from genomic DNA libraries. The black line and the corresponding *y*-axis on the left depict the total level of cytosine methylation in all combined contexts (CG, CHG, or CHH). The orange line and the corresponding *y*-axis on the right depict normalized read coverage. WGBS data used to produce this figure were obtained from line 1 replicate 2 from (Schmitz et al., 2011). Genomic DNA sequencing data used to produce this figure were obtained from sample 119 in (Becker et al., 2011).

**FIGURE 3:**
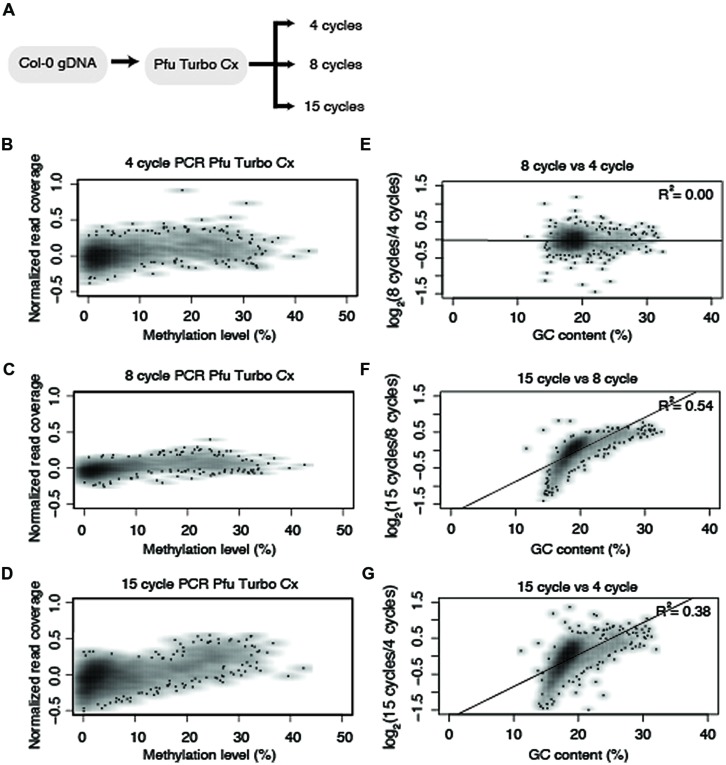
**Polymerase chain reaction (PCR) cycle number influences enrichment bias. (A)** Diagram of the experimental design for testing the effect PCR cycle number on representation of methylated DNA. Normalized read counts versus methylation levels of 10 kb fragments from chromosome five for **(B)** 4 cycles, **(C)** 8 cycles, and **(D)** 15 cycles of PCR amplification using Pfu Turbo Cx. **(E–G)** Normalized read numbers for each 10 kb window were compared between different PCR cycle numbers and correlation scores were determined as indicated in the upper right hand of the plot. All R^2^ values reported reflect Pearson correlation coefficients.

**FIGURE 4:**
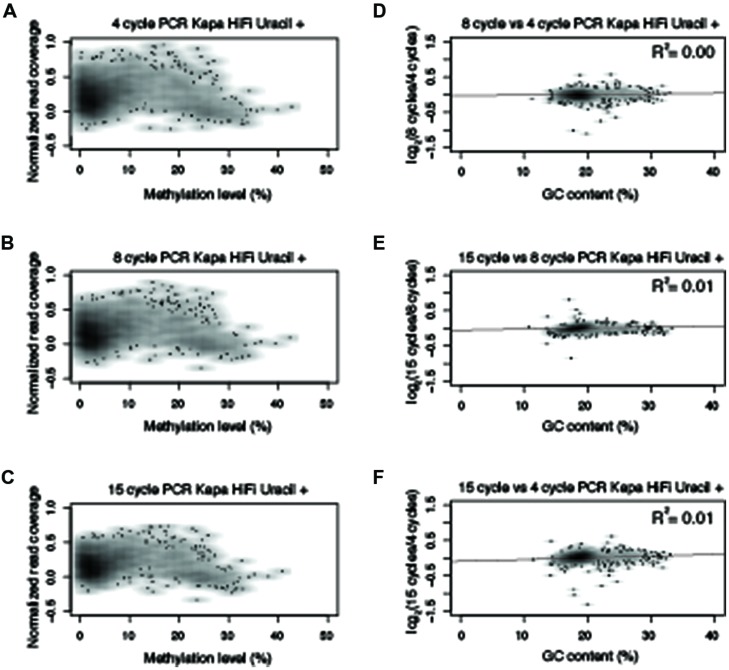
**Polymerase chain reaction amplification bias observed with Kapa HiFi Uracil +.** Normalized read counts versus methylation levels for 10 kb windows from chromosome five for **(A–C)** Kapa HiFi Uracil + from 4-, 8-, and 15-cycles of PCR. **(D–F)** Normalized read numbers for each 10 kb window were compared between different PCR cycle numbers and correlation scores were determined as indicated in the upper right hand of the plot. All R^2^ values reported reflect Pearson correlation coefficients.

**FIGURE 5:**
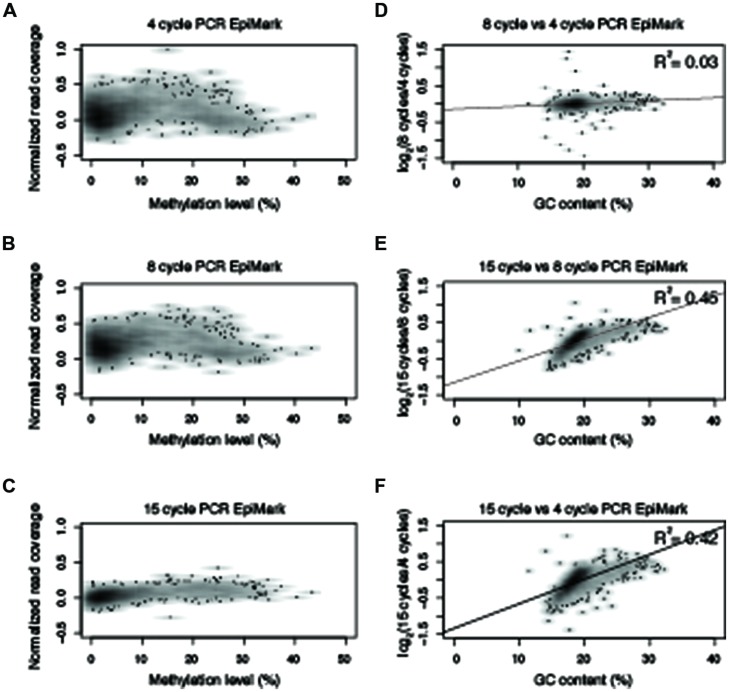
**Polymerase chain reaction amplification bias observed with NEB EpiMark.** Normalized read counts versus methylation levels for 10 kb windows from chromosome five for **(A–C)** NEB EpiMark from 4-, 8-, and 15-cycles of PCR. **(D–F)** Normalized read numbers for each 10 kb window were compared between different PCR cycle numbers and correlation scores were determined as indicated in the upper right hand of the plot. All R^2^ values reported reflect Pearson correlation coefficients.

**FIGURE 6:**
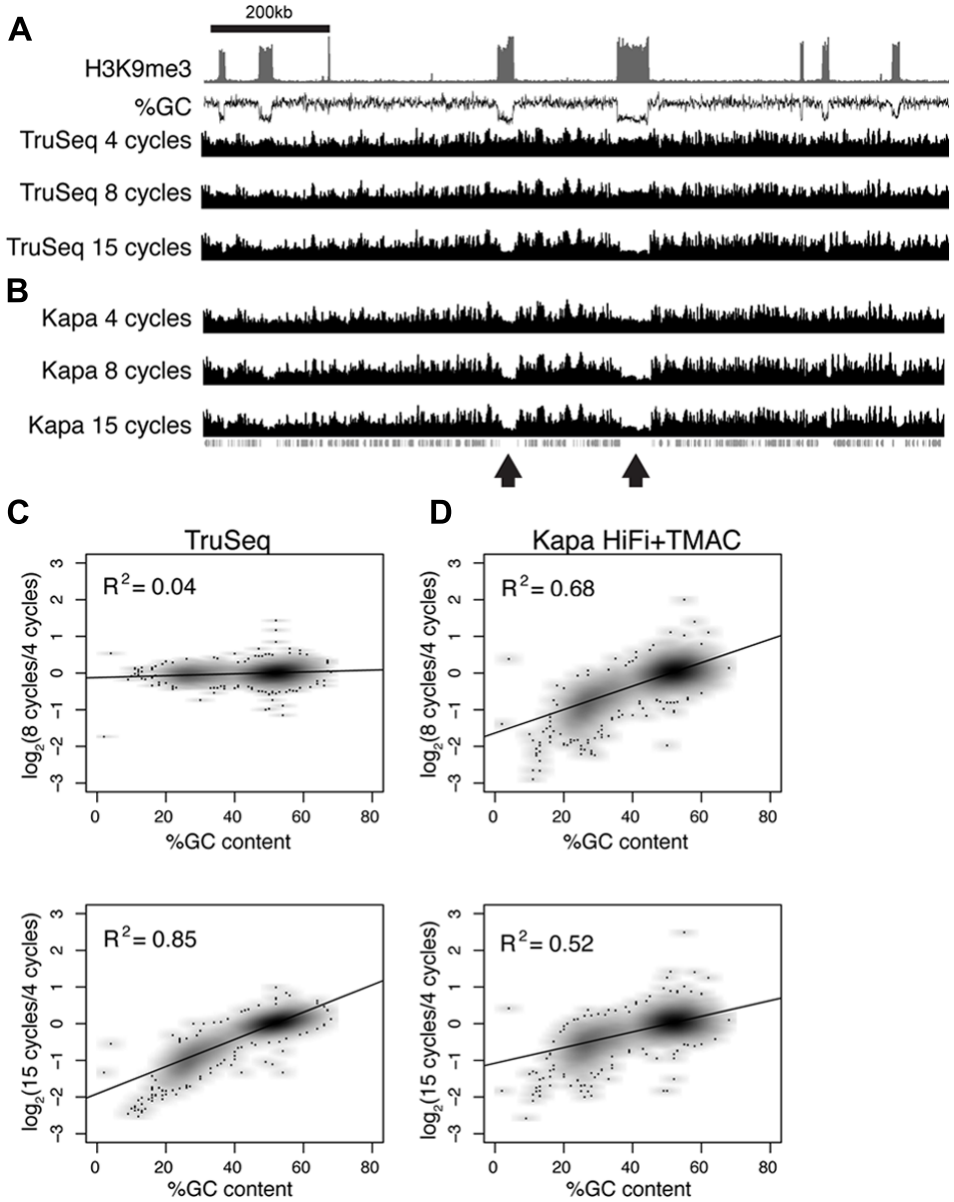
**Polymerase chain reaction amplification bias in *Neurospora crassa* heterochromatin domains **(A–B)** Sequence coverage is shown across a 1.2 Mb domain of the *Neurospora* genome (chromosome 1: 200–1400 kb).** Enrichment of tri-methyl H3 K9 (H3K9me3; Sasaki et al., 2014) and GC-content (%GC) are plotted to indicate the position of AT-rich heterochromatin domains. Data are shown for libraries prepared with Illumina PCR master mix **(A)** or Kapa HiFi polymerase + TMAC **(B)** and amplified for 4, 8, and 15 cycles, as indicated. The positions of genes are plotted beneath the coverage tracks. **(C–D)** Normalized read coverage and GC-content was calculated for 1 kb windows across the entire *Neurospora* genome for libraries prepared using different conditions. Log_2_ values obtained by comparing normalized read coverage after 4 and 8 cycles **(top)** or 4 and 15 cycles **(bottom)** are plotted on the *y*-axis. %GC is plotted on the *x*-axis. Results for TruSeq master mix and Kapa HiFi polymerase are shown in panels **(C,D)**, respectively.

For **Figures 1D–F** normalized read coverage was calculated by the total number of reads in the DMR (Differentially Methylated Region) divided by the total number of reads sequenced from the library. This value was then multiplied by 10^5^. For **Figure 1G**, normalized read coverage was calculated as follows: genome sequencing reads from previously published *A. thaliana* Col-0 data (Becker et al., 2011) were used to determine a baseline level of mappability by the DNA that had not undergone sodium bisulfite treatment. In this case, all cytosines were converted to thymines in the FASTQ files and then the genome sequencing data set was used in the same analysis pipeline as the one used to map and determine methylation levels for sodium bisulfite-treated libraries. *A. thaliana* mutation accumulation line 1 replicate 2 was selected as bisulfite sequencing sample (Schmitz et al., 2011). In each 10 kb window, read numbers were counted and then divided by the number of mapped reads in the first tile to normalize them to the same scale. Normalized read coverage was then computed by subtracting the normalized number of reads present in the genomic DNA sample from the normalized number of reads in the bisulfite sequencing sample. 5% outliers were identified, as described in the Calculation of correlation coefficients section, and removed before computing linear regression (Cook and Weisberg, 1982). For **Figure 2B**, the window size was set to 100 kb, the enrichment of mapped reads for each tile was computed using the same method as described for 1 G. For **Figures 3–5**, the methylation levels from **Figure 1G** were used to set a fixed horizontal axis. Therefore, data points were only able to move in a vertical direction depending on normalized read coverage. This method was used because higher read coverage resulted in higher methylation levels, which made it challenging to accurately reveal and compare the trends in each figure. For **Figure 6**, to calculate normalized read coverage for *Neurospora*, the genome was divided into 1 kb windows using bedtools, and a gtf annotation file was constructed using custom scripts. Cuffdiff software (Trapnell et al., 2013) and the custom annotation file were used to calculate the enrichment ratios at each 1 kb window. %GC was determined using a custom script.

### DATA ACCESS

The data generated for this study have been deposited in the Gene Expression Omnibus^1^ and are accessible through accession number GSE58217. For WGBS data in **Figures 1** and **2** data were downloaded from the National Center for Biotechnology Information (NCBI) – Sequence Read Archive (SRA)^2^ and for genomic DNA sequence used in **Figure 2** data were downloaded from ().

## RESULTS AND DISCUSSIONS

### METHYLATED DNA IS OVER-REPRESENTED IN BISULFITE SEQUENCING DATA

Our previous studies that applied MethylC-seq (a WBGS method) on a population of *A. thaliana* MA lines (Shaw et al., 2000) revealed the existence of spontaneous epialleles (Schmitz et al., 2011; **Figures 1A–C**). These differentially methylated regions (DMRs) of the population have identical underlying DNA sequences as revealed by whole genome sequencing (Ossowski et al., 2010), but vary significantly in DNA methylation levels (**Figures 1A–C**). Further examination of the underlying data revealed that reads from highly methylated DMRs were over-represented compared to unmethylated regions after bisulfite conversion and DNA sequencing (**Figures 1D–F**). In total, eight different MA lines were sequenced in duplicate for a total of 16 samples. In every case, there was a positive correlation between the methylation level of the DMR and the normalized number of aligned sequenced reads (**Figures 1D–F**). Similarly, a chromosome-wide test revealed a strong positive correlation between DNA methylation levels and normalized (aligned) read counts from WGBS data (**Figure 1G**; R^2^ = 0.55).

### GC CONTENT INFLUENCES ENRICHMENT OF HIGHLY METHYLATED DNA

Although the sequences within DMRs are identical, GC contents were significantly different after sodium bisulfite conversion. Therefore, the possibility exists that the underlying GC content likely influences the enrichment bias of methylated DNA. To further explore this question, the GC content throughout chromosome five was determined prior to and post sodium bisulfite conversion (**Figure 2A**). Post bisulfite treatment, the GC content is significantly reduced by approximately 50%, which is expected as most of the cytosines are unmethylated and are converted to uracils and ultimately thymines after PCR. The lowest percentage reduction in GC content is observed in the pericentromeric region of the chromosome (**Figure 2A**) where the highest density of methylation has been observed (**Figure 2B**; Cokus et al., 2008; Lister et al., 2008). To empirically test if sodium bisulfite-treated and amplified DNA is preferentially biased toward highly methylated reads, both WBGS and genomic DNA sequencing data from wild-type Col-0 were aligned using the same parameters. The read enrichment was calculated and plotted along chromosome five, which revealed an enrichment of WGBS reads compared to genomic DNA sequencing reads that paralleled DNA methylation levels along the chromosome (**Figure 2B**). Collectively, these data suggest that highly methylated sequences, which retain high GC content after bisulfite conversion and PCR amplification, are preferentially enriched in WGBS data compared to unmethylated sequences.

### NUMBER OF PCR CYCLES INFLUENCES ENRICHMENT OF HIGHLY METHYLATED DNA

The introduction of altered GC content occurs during bisulfite treatment and subsequent PCR amplification. Bisulfite-converted DNA is rich with uracils, which impairs the ability of most DNA polymerases to efficiently amplify these fragments. The B-type DNA polymerase from *Pyrococcus furiosus* is able to polymerize uracil-rich DNA because it binds to deaminated bases (Horvath and Vertessy, 2010). This enzyme was engineered to polymerize uracil-rich DNA by mutating a single amino acid that blocks recognition of the deaminated bases (Horvath and Vertessy, 2010). These types of engineered uracil-tolerant enzymes are critical for single-base resolution studies of DNA methylation and as a result, a number of commercially available enzymes have been engineered to amplify uracil-containing templates. We performed a test on the Pfu Turbo Cx enzyme with biological replicates (Supplementary Table 1) using three different cycles (4, 8, and 15 cycles) of PCR (**Figure 3A**) to assess the degree to which a uracil-insensitive enzyme is prone to an amplification bias of methylated DNA. A comparison between 4, 8, and 15 PCR cycles revealed that 15 cycles results in an exacerbation in the preferential enrichment of methylated DNA (**Figures 3B–G**).

### COMPARATIVE ANALYSIS OF COMMERCIALLY AVAILABLE URACIL-INSENSITIVE ENZYMES

Based on the results from **Figure 3**, an experiment was designed to test two additional commonly used commercially available enzymes in biological replicates (Supplementary Table 1) for their ability to amplify bisulfite-converted DNA (Kapa HiFi Uracil + and EpiMark). Upon constructing the MethylC-seq libraries the concentration of the samples were determined using a Qubit. This revealed that the total library yield for the 4-cycle/8-cycle PCR was similar between the three enzymes tested, but for the 15-cycle PCR, the KAPA HiFi Uracil + enzyme yielded significantly more total library (**Table 1**). Regardless, the total amount of library yielded in all experiments is more than adequate to sequence to sufficient depth. Next, the methylation levels were plotted against normalized read counts and as observed with the Pfu Turbo Cx enzyme in **Figures 1** and **2**, the other two tested enzymes also preferentially enrich methylated DNA, although to a lesser extent (**Figures 4** and **5**). However, both of these enzymes are sensitive to highly methylated sequences as the normalized read numbers started to decrease with higher methylation levels compared to intermediate methylation levels (**Figures 4** and **5**). Interestingly, between the 4, 8, and 15 cycle experiments for the Kapa HiFi Uracil + enzyme, extremely high GC content regions did not result in an increase in amplification bias of DNA like it does with both the Pfu Turbo Cx and EpiMark enzymes (**Figures 3–5**), which would indicate this enzyme is likely preferred for most WGBS applications. Interestingly, the EpiMark enzyme revealed that at 15 PCR cycles a strong bias was associated with increasing GC content, but not with increasing levels of DNA methylation compared to the 4- and 8-cycle reactions (**Figures 5A–F**). Therefore, both PCR cycles and enzymes selected for amplification can influence enrichment of highly methylated DNA in sequencing data.

**Table 1:** Sample name, cycle number and total library yield for each WGBS library are reported.

Sample name	Cycle number	Total library yield (ng/ul) replicate 1	Total library yield (ng/ul) replicate 2	Correlation coefficient between biological replicates
Kapa HiFi U+	4	0.44	0.38	0.9167678
Pfu Turbo Cx	4	0.49	0.39	0.5800957
NEB EpiMark	4	0.59	0.25	0.8791214
Kapa HiFi U+	8	2.32	1.63	0.980213
Pfu Turbo Cx	8	1.45	1.03	0.8921665
NEB EpiMark	8	2.64	1.89	0.9589284
Kapa HiFi U+	15	80.8	62.4	0.9677855
Pfu Turbo Cx	15	40.2	36.1	0.9730408
NEB EpiMark	15	52.6	39.2	0.8796196

*The correlation coefficient was calculated based on the normalized reads coverage between the biological replicates.*

### AT-RICH SEQUENCES ARE DEPLETED FROM DNA SEQUENCING LIBRARIES

ChIP-seq experiments carried out with the filamentous fungus *Neurospora crassa* also revealed a bias against AT-rich DNA sequences, demonstrating reduced coverage at AT-rich DNA is not restricted to uracil-tolerant polymerases. *Neurospora* heterochromatin domains have an average GC content of 30% compared to an average GC content of 51.5% for genes (**Figure 6**). ChIP input DNA was used to prepare Illumina sequencing libraries using two different polymerases and PCR was performed for 4, 8, and 15 cycles. Libraries prepared using a TruSeq PCR master-mix revealed equal representation of GC-rich and AT-rich regions after 4 and 8 cycles of PCR, but a clear amplification bias was observed after 15 cycles of PCR (**Figures 6A,C**). It was reported previously that the Kapa HiFi polymerase, a distinct enzyme from the Kapa HiFi Uracil + polymerase used throughout the rest of the manuscript, efficiently amplified AT-rich DNA when supplemented with 60 mM TMAC (Oyola et al., 2012). However, our experiments with *Neurospora* DNA revealed a significant underrepresentation of AT-rich DNA after only 8 cycles of PCR using the KAPA HiFi DNA polymerase + TMAC (**Figures 6B,D**). Therefore, the DNA polymerase used for PCR amplification and the number of PCR cycles selected can affect amplification of genomic DNA that contains large disparities in GC content.

## CONCLUSION

Whole-genome bisulfite sequencing is an excellent method for determining base resolution DNA methylomes and although in most genomes studied to date, it has been able to determine methylation levels for >90% of cytosines, it does have some limitations. In this study, we show that there exists a strong amplification bias of methylated DNA compared to unmethylated sequences and that this bias can be affected by PCR cycle number and the enzyme used for PCR amplification. Interestingly, this change in amplification bias is not observed with the Kapa HiFi Uracil + enzyme in comparison to the others tested. This observation suggests that the high PCR cycle number that is necessary to construct a sequenceable library for low input samples such as RRBS (Gu et al., 2011), single cells, sequence-capture or FFPE samples can be achieved without introducing major artifacts from the number of PCR cycles selected. Therefore, although an amplification bias will occur at these highly methylated sequences, in these types of libraries, it will not grow in strength to the point that it depletes lower methylated sequences from produced sequencing data. This effect is not true for all enzymes used in amplification of bisulfite converted DNA as the Pfu Turbo Cx enzyme resulted in a significant over-enrichment of reads associated with methylated DNA with increasing cycle number. Furthermore, even tests on genomic DNA from *Neurospora* revealed significant variation between different commercially available enzymes and selected PCR cycle number. To improve the quality and to reduce to bias associated with WGBS data it is recommended to start higher concentrations of genomic DNA when possible, limit the number of PCR cycles and optimize the polymerase used for amplification of the library.

In general, it is difficult to precisely quantify the effect of this observed bias in WGBS data on percent methylation calculations. As we show here, bias depends on polymerase choice and PCR cycle number. We expect that other factors can also affect bias, not only the methylation level, which itself can be affected by biological and technical variation, but also factors like raw sequence read length, sequencing errors, efficiency of sodium bisulfite conversion, sequence structure itself, etc. In order to develop computational tools that correct for bias, additional experiments are needed to define how these factors contribute to the observed bias in WGBS data. We note that the bias introduced using current protocols is likely to influence DMR analysis. In particular, loci that are heterozygous for methylation state could be underrepresented by DMR finding algorithms.

The sodium bisulfite conversion and PCR amplification steps are impossible to remove from WGBS library preparation and these are the steps where a bias is introduced. In fact, efforts to correct for amplification bias in targeted bisulfite sequencing of genomic regions have been successfully performed by using a calibration method that requires a parallel analysis of input samples with known methylation levels (Moskalev et al., 2011). Additionally, RainDance Technology has also proved useful for correction of amplification bias in targeted bisulfite PCR (Komori et al., 2011), but it is unknown if this correction will be observed on WGBS libraries. Regardless, both of these methods are not feasible alternatives for routine correction of the observed amplification bias in WGBS, as they are technically cumbersome compared to current methods and they are cost prohibitive. This bias will be most pronounced in the study of allele-specific methylation. The results of this study make it clear that PCR enzyme and the PCR amplification cycle used can influence determined methylation levels, which undoubtedly could affect results of previously published studies that were not aware of these biases. Fortunately, most experiments that use this approach can be corrected for this bias by using SNP data in the bisulfite-sequencing reads. Caution should be taken when calculating methylation levels at loci with heterozygous methylation states, especially when experiments study regions that do not have SNPs or do not take into consideration SNPs linked to bisulfite-sequencing reads. Caution should also be taken when using platforms that do not detect SNPs. For example, many microarray platforms have been designed to detect DNA methylation states and although PCR is not generally used to amplify bisulfite-converted DNA, the use of Whole Genome Amplification (WGA) methods will still introduce a bias based on the GC content of DNA sequences (Bredel et al., 2005; Arriola et al., 2007). Because WGBS is primarily used on species with published reference genomes, it may be possible to predict this amplification bias based on GC content and the range of potential methylation levels at these regions. Therefore, future efforts to computationally model and predict expected read coverage for regions will be advantageous for studies where SNP information is not available or present at specific regions of interest.

## Conflict of Interest Statement

The authors declare that the research was conducted in the absence of any commercial or financial relationships that could be construed as a potential conflict of interest.

## Footnotes

1 http://www.ncbi.nlm.nih.gov/geo/

2 http://www.ncbi.nlm.nih.gov/sra/SRX096372

## References

[B1] Aird D. Ross M. G. Chen W. S. Danielsson M. Fennell T. Russ C. (2011). Analyzing and minimizing PCR amplification bias in Illumina sequencing libraries. *Genome Biol.* 12 R18. 10.1186/gb-2011-12-2-r18 PMC3188800 21338519

[B2] Arriola E. Lambros M. B. Jones C. Dexter T. Mackay A. Tan D. S. (2007). Evaluation of Phi29-based whole-genome amplification for microarray-based comparative genomic hybridisation. *Lab. Invest.* 87 75–83. 10.1038/labinvest.3700495 17170740

[B3] Becker C. Hagmann J. Muller J. Koenig D. Stegle O. Borgwardt K. (2011). Spontaneous epigenetic variation in the *Arabidopsis thaliana* methylome. *Nature* 480 245–249. 10.1038/nature10555 22057020

[B4] Benjamini Y. Speed T. P. (2012). Summarizing and correcting the GC content bias in high-throughput sequencing. *Nucleic Acids Res.* 40 e72. 10.1093/nar/gks001 PMC3378858 22323520

[B5] Bernstein B. E. Stamatoyannopoulos J. A. Costello J. F. Ren B. Milosavljevic A. Meissner A. (2010). The NIH roadmap epigenomics mapping consortium. *Nat. Biotechnol.* 28 1045–1048. 10.1038/nbt1010-1045 20944595 PMC3607281

[B6] Booth M. J. Branco M. R. Ficz G. Oxley D. Krueger F. Reik W. (2012). Quantitative sequencing of 5-methylcytosine and 5-hydroxymethylcytosine at single-base resolution. *Science* 336 934–937. 10.1126/science.1220671 22539555

[B7] Booth M. J. Marsico G. Bachman M. Beraldi D. (2014). Quantitative sequencing of 5-formylcytosine in DNA at single-base resolution. *Nat. Chem.* 6 435–440. 10.1038/NCHEM.1893 24755596 PMC4188980

[B8] Bredel M. Bredel C. Juric D. Kim Y. Vogel H. Harsh G. R. (2005). Amplification of whole tumor genomes and gene-by-gene mapping of genomic aberrations from limited sources of fresh-frozen and paraffin-embedded DNA. *J. Mol. Diagn.* 7 171–182. 10.1016/S1525-1578(10)60543-0 15858140 PMC1867518

[B9] Calarco J. P. Borges F. Donoghue M. T. Van Ex F. Jullien P. E. Lopes T. (2012). Reprogramming of DNA methylation in pollen guides epigenetic inheritance via small RNA. *Cell* 151 194–205. 10.1016/j.cell.2012.09.001 23000270 PMC3697483

[B10] Clark S. J. Harrison J. Paul C. L. Frommer M. (1994). High sensitivity mapping of methylated cytosines. *Nucleic Acids Res.* 22 2990–2997. 10.1093/nar/22.15.2990 8065911 PMC310266

[B11] Clark S. J. Statham A. Stirzaker C. Molloy P. L. Frommer M. (2006). DNA methylation: bisulphite modification and analysis. *Nat. Protoc.* 1 2353–2364 17406479 10.1038/nprot.2006.324

[B12] Cokus S. J. Feng S. Zhang X. Chen Z. Merriman B. Haudenschild C. D. (2008). Shotgun bisulphite sequencing of the *Arabidopsis* genome reveals DNA methylation patterning. *Nature* 452 215–219. 10.1038/nature06745 18278030 PMC2377394

[B13] Cook R. D. Weisberg S. (1982). *Residuals and Influence in Regression*. New York: Chapman and Hall.

[B14] Creasey K. M. Zhai J. Borges F. Van Ex F. Regulski M. Meyers B. C. (2014). miRNAs trigger widespread epigenetically activated siRNAs from transposons in *Arabidopsis*. *Nature* 508 411–415. 10.1038/nature13069 24670663 PMC4074602

[B15] Frommer M. Mcdonald L. E. Millar D. S. Collis C. M. Watt F. Grigg G. W. (1992). A genomic sequencing protocol that yields a positive display of 5-methylcytosine residues in individual DNA strands. *Proc. Natl. Acad. Sci. U.S.A.* 89 1827–1831. 10.1073/pnas.89.5.1827 1542678 PMC48546

[B16] Gehring M. Bubb K. L. Henikoff S. (2009). Extensive demethylation of repetitive elements during seed development underlies gene imprinting. *Science* 324 1447–1451. 10.1126/science.1171609 19520961 PMC2886585

[B17] Gent J. I. Ellis N. A. Guo L. Harkess A. E. Yao Y. Zhang X. (2013). CHH islands: de novo DNA methylation in near-gene chromatin regulation in maize. *Genome Res.* 23 628–637. 10.1101/gr.146985.112 23269663 PMC3613580

[B18] Gu H. Smith Z. D. Bock C. Boyle P. Gnirke A. Meissner A. (2011). Preparation of reduced representation bisulfite sequencing libraries for genome-scale DNA methylation profiling. *Nat. Protoc.* 6 468–481. 10.1038/nprot.2010.190 21412275

[B19] Hayatsu H. (1976). Bisulfite modification of nucleic acids and their constituents. *Prog. Nucleic Acid Res. Mol. Biol.* 16 75–124 2948 10.1016/s0079-6603(08)60756-4

[B20] Hayatsu H. Wataya Y. Kai K. Iida S. (1970). Reaction of sodium bisulfite with uracil, cytosine, and their derivatives. *Biochemistry* 9 2858–2865. 10.1021/bi00816a016 5459538

[B21] Horvath A. Vertessy B. G. (2010). A one-step method for quantitative determination of uracil in DNA by real-time PCR. *Nucleic Acids Res.* 38 e196. 10.1093/nar/gkq815 PMC2995087 20864450

[B22] Hsieh T. F. Ibarra C. A. Silva P. Zemach A. Eshed-Williams L. Fischer R. L. (2009). Genome-wide demethylation of *Arabidopsis* endosperm. *Science* 324 1451–1454. 10.1126/science.1172417 19520962 PMC4044190

[B23] Ibarra C. A. Feng X. Schoft V. K. Hsieh T. F. Uzawa R. Rodrigues J. A. (2012). Active DNA demethylation in plant companion cells reinforces transposon methylation in gametes. *Science* 337 1360–1364. 10.1126/science.1224839 22984074 PMC4034762

[B24] Jiang L. Zhang J. Wang J. J. Wang L. Zhang L. Li G. (2013). Sperm, but not oocyte, DNA methylome is inherited by zebrafish early embryos. *Cell* 153 773–784. 10.1016/j.cell.2013.04.041 23663777 PMC4081501

[B25] Kobayashi H. Sakurai T. Miura F. Imai M. Mochiduki K. Yanagisawa E. (2013). High-resolution DNA methylome analysis of primordial germ cells identifies gender-specific reprogramming in mice. *Genome Res.* 23 616–627. 10.1101/gr.148023.112 23410886 PMC3613579

[B26] Komori H. K. Lamere S. A. Torkamani A. Hart G. T. Kotsopoulos S. Warner J. (2011). Application of microdroplet PCR for large-scale targeted bisulfite sequencing. *Genome Res.* 21 1738–1745. 10.1101/gr.116863.110 21757609 PMC3202290

[B27] Kozarewa I. Ning Z. Quail M. A. Sanders M. J. Berriman M. Turner D. J. (2009). Amplification-free Illumina sequencing-library preparation facilitates improved mapping and assembly of (G+C)-biased genomes. *Nat. Methods* 6 291–295. 10.1038/nmeth.1311 19287394 PMC2664327

[B28] Lister R. O’malley R. C. Tonti-Filippini J. Gregory B. D. Berry C. C. Millar A. H. (2008). Highly integrated single-base resolution maps of the epigenome in *Arabidopsis*. *Cell* 133 523–536. 10.1016/j.cell.2008.03.029. 18423832 PMC2723732

[B29] Lister R. Pelizzola M. Dowen R. H. Hawkins R. D. Hon G. Tonti-Filippini J. (2009). Human DNA methylomes at base resolution show widespread epigenomic differences. *Nature* 462 315–322. 10.1038/nature08514 19829295 PMC2857523

[B30] Lister R. Pelizzola M. Kida Y. S. Hawkins R. D. Nery J. R. Hon G. (2011). Hotspots of aberrant epigenomic reprogramming in human induced pluripotent stem cells. *Nature* 471 68–73. 10.1038/nature09798 21289626 PMC3100360

[B31] McCluskey K. Wiest A. Plamann M. (2010). The Fungal Genetics Stock Center: a repository for 50 years of fungal genetics research. *J. Biosci.* 35 119–126 20413916 10.1007/s12038-010-0014-6

[B32] Meissner A. Gnirke A. Bell G. W. Ramsahoye B. Lander E. S. Jaenisch R. (2005). Reduced representation bisulfite sequencing for comparative high-resolution DNA methylation analysis. *Nucleic Acids Res.* 33 5868–5877. 10.1093/nar/gki901 16224102 PMC1258174

[B33] Moskalev E. A. Zavgorodnij M. G. Majorova S. P. Vorobjev I. A. Jandaghi P. Bure I. V. (2011). Correction of PCR-bias in quantitative DNA methylation studies by means of cubic polynomial regression. *Nucleic Acids Res.* 39 e77. 10.1093/nar/gkr213 PMC3113592 21486748

[B34] Orozco L. D. Rubbi L. Martin L. J. Fang F. Hormozdiari F. Che N. (2014). Intergenerational genomic DNA methylation patterns in mouse hybrid strains. *Genome Biol.* 15 R68. 10.1186/gb-2014-15-5-r68 PMC4076608 24887417

[B35] Ossowski S. Schneeberger K. Lucas-Lledo J. I. Warthmann N. Clark R. M. Shaw R. G. (2010). The rate and molecular spectrum of spontaneous mutations in *Arabidopsis thaliana*. *Science* 327 92–94. 10.1126/science.1180677 20044577 PMC3878865

[B36] Oyola S. O. Otto T. D. Gu Y. Maslen G. Manske M. Campino S. (2012). Optimizing Illumina next-generation sequencing library preparation for extremely AT-biased genomes. *BMC Genomics* 13:1. 10.1186/1471-2164-13-11 PMC3312816 22214261

[B37] Sasaki T. Lynch K. L. Mueller C. V. Friedman S. Freitag M. Lewis Z. A. (2014). Heterochromatin controls gammaH2A localization in *Neurospora crassa*. *Eukaryot. Cell 1* 3 990–1000. 10.1128/EC.00117-114 PMC4135800 24879124

[B38] Schmitz R. J. He Y. Valdes-Lopez O. Khan S. M. Joshi T. Urich M. A. (2013a). Epigenome-wide inheritance of cytosine methylation variants in a recombinant inbred population. *Genome Res.* 23 1663–1674. 10.1101/gr.152538.112 23739894 PMC3787263

[B39] Schmitz R. J. Schultz M. D. Urich M. A. Nery J. R. Pelizzola M. Libiger O. (2013b). Patterns of population epigenomic diversity. *Nature* 495 193–198. 10.1038/nature11968 23467092 PMC3798000

[B40] Schmitz R. J. Schultz M. D. Lewsey M. G. O’malley R. C. Urich M. A. Libiger O. (2011). Transgenerational epigenetic instability is a source of novel methylation variants. *Science* 334 369–373. 10.1126/science.1212959 21921155 PMC3210014

[B41] Schultz M. D. Schmitz R. J. Ecker J. R. (2012). “Leveling” the playing field for analyses of single-base resolution DNA methylomes. *Trends Genet.* 28 583–585. 10.1016/j.tig.2012.10.012 23131467 PMC3523709

[B42] Seisenberger S. Andrews S. Krueger F. Arand J. Walter J. Santos F. (2012). The dynamics of genome-wide DNA methylation reprogramming in mouse primordial germ cells. *Mol. Cell* 48 849–862. 10.1016/j.molcel.2012.11.001 23219530 PMC3533687

[B43] Shapiro R. Braverman B. Louis J. B. Servis R. E. (1973). Nucleic acid reactivity and conformation. II. Reaction of cytosine and uracil with sodium bisulfite. *J. Biol. Chem.* 248 4060–4064 4736082

[B44] Shapiro R. Difate V. Welcher M. (1974). Deamination of cytosine derivatives by bisulfite. Mechanism of the reaction. *J. Am. Chem. Soc.*. 96 906–912 4814744 10.1021/ja00810a043

[B45] Shaw R. G. Byers D. L. Darmo E. (2000). Spontaneous mutational effects on reproductive traits of *Arabidopsis thaliana*. *Genetics* 155 369–378 10790410 10.1093/genetics/155.1.369 PMC1461073

[B46] Shirane K. Toh H. Kobayashi H. Miura F. Chiba H. Ito T. (2013). Mouse oocyte methylomes at base resolution reveal genome-wide accumulation of non-CpG methylation and role of DNA methyltransferases. *PLoS Genet.* 9:e1003439. 10.1371/journal.pgen.1003439 PMC3630097 23637617

[B47] Slotkin R. K. Vaughn M. Borges F. Tanurdzic M. Becker J. D. Feijo J. A. (2009). Epigenetic reprogramming and small RNA silencing of transposable elements in pollen. *Cell* 136 461–472. 10.1016/j.cell.2008.12.038. 19203581 PMC2661848

[B48] Stadler M. B. Murr R. Burger L. Ivanek R. Lienert F. Scholer A. (2011). DNA-binding factors shape the mouse methylome at distal regulatory regions. *Nature* 480 490–495. 10.1038/nature10716 22170606

[B49] Trapnell C. Hendrickson D. G. Sauvageau M. Goff L. Rinn J. L. Pachter L. (2013). Differential analysis of gene regulation at transcript resolution with RNA-seq. *Nat. Biotechnol.* 31 46–53. 10.1038/nbt.2450 23222703 PMC3869392

[B50] Urich M. A. Nery J. R. Lister R. Schmitz R. J. Ecker J. R. (2014). MethylC-seq: base resolution whole genome bisulfite sequencing library preparation. *Nat. Protoc.* (in press) 10.1038/nprot.2014.114 PMC4465251 25692984

[B51] Warnecke P. M. Stirzaker C. Melki J. R. Millar D. S. Paul C. L. Clark S. J. (1997). Detection and measurement of PCR bias in quantitative methylation analysis of bisulphite-treated DNA. *Nucleic Acids Res.* 25 4422–4426 9336479 10.1093/nar/25.21.4422 PMC147052

[B52] Xie W. Barr C. L. Kim A. Yue F. Lee A. Y. Eubanks J. (2012). Base-resolution analyses of sequence and parent-of-origin dependent DNA methylation in the mouse genome. *Cell* 148 816–831. 10.1016/j.cell.2011.12.035 22341451 PMC3343639

[B53] Yu M. Hon G. C. Szulwach K. E. Song C. X. Zhang L. Kim A. (2012). Base-resolution analysis of 5-hydroxymethylcytosine in the mammalian genome. *Cell* 149 1368–1380. 10.1016/j.cell.2012.04.027 22608086 PMC3589129

[B54] Zemach A. Kim M. Y. Silva P. Rodrigues J. A. Dotson B. Brooks M. D. (2010). Local DNA hypomethylation activates genes in rice endosperm. *Proc. Natl. Acad. Sci. U.S.A.* 107 18729–18734. 10.1073/pnas.1009695107 20937895 PMC2972920

